# Expression characteristics of *CsPG23* in citrus and analysis of its interacting protein

**DOI:** 10.1080/15592324.2025.2508418

**Published:** 2025-05-22

**Authors:** Qing He, Xiao He

**Affiliations:** School of Medical Technology, Chongqing Three Gorges Medical College, Chongqing, People’s Republic of China

**Keywords:** Citrus, hairy roots, CsPG23, CsAGD8

## Abstract

Exploring the resistance genes of citrus to Huanglongbing (HLB) is the foundation and key to citrus disease-resistant breeding. Through the analysis of comparative transcriptome data, we identified six cell wall degradation genes that respond to citrus infection with *Ca*Las. We selected one of the genes with high differential expression levels and cloned it, naming it *CsPG23*. The subcellular localization results of tobacco indicated that the *CsPG23* protein is localized in the nucleus, cytoplasm, and cell membrane. Real-time fluorescence quantitative PCR (RT-qPCR) analysis showed that the expression of *CsPG23* is related to variety tolerance, tissue location, and symptom development. In addition, we constructed overexpression and silencing vectors for *CsPG23* and obtained *CsPG23* silencing plants, overexpression and silencing hairy roots, and analyzed the expression characteristics of *CsPG23* in response to SA, JA, MeSA and H_2_O_2_ induction through RT-qPCR. Using Protein–Protein Interaction (PPI) to predict and screen for a citrus protein CsAGD8 that may interact with CsPG23, and preliminarily verifying its interaction with CsPG23 protein through Yeast Two-hybrid (Y2H). We constructed overexpression and silencing vectors for *CsAGD8* and obtained *CsAGD8* overexpression and silencing hairy roots. In summary, it is indicated that CsPG23 may interact with CsAGD8 in response to *Ca*Las infection.

## Introduction

Huanglongbing (HLB) is a bacterial disease that poses a serious threat to the citrus industry. The pathogen of this disease is mainly gram-negative bacterium, which can be divided into three types: “*Candidatus* liberibacter asiaticus” (*Ca*Las), “*Candidatus* liberibacter africanus” (*Ca*Laf), and “*Candidatus* liberibacter americanus” (*Ca*Lam).^1^ Among them, *Ca*Las have the widest distribution globally and the strongest pathogenicity, becoming the main challenge for prevention and control^2^). The transmission path of this disease mainly depends on citrus psyllids and grafting.^3^ Once infected, it will have irreversible effects on the growth of citrus trees. At present, there are no effective treatment measures or disease-resistant varieties for citrus HLB.^4^ Therefore, how to strengthen the prevention and control of this disease has become an urgent problem to be solved in the citrus industry.^5^ In this context, it is particularly important to explore and utilize the resistance genes of citrus plants, which not only provides new ideas for preventing and controlling HLB, but also provides strong support for improving the sustainable development capacity of the citrus industry.

The length of the transcription unit coding region (CDS) and sequence of the polygalacturonase (PG) gene vary significantly depending on species and gene family members. In the PG gene family of *Akebia trifoliata*, the CDS length range is between 1200 and 1500 bp. For example, the CDS of key genes such as *AKTpg25* is about 1380 bp, corresponding to approximately 460 amino acids.^6^ The CDS length of the PG gene in *Populus varies* greatly, with some members reaching over 2000 bp. For example, some members of the PtPG gene family have CDS exceeding 2000 bp.^7^ Each genome may only contain one copy of the PG gene. After sorting and analyzing all eukaryotic and prokaryotic PG protein sequences, four conserved domains were found.^8^ Among them, cysteine residues in domain III are believed to be related to plant disease resistance responses. The positions of these cysteine residues have good conservation in different protein sequences, indicating that they play important roles in protein function.^9^

PG, as a cell wall binding protein, plays an important role in plant growth, development, and physiological processes, involving multiple aspects such as fruit ripening, leaf and flower shedding, pod cracking, pollen maturation, immune defense, and plant interaction with the external environment.^10^ Inhibition of PG activity in genetically modified tomato fruits can slow down the degradation rate of pectin. When pathogenic bacteria infect plants, PG enzymes in the plant cell wall will exert their hydrolysis effect, actively decompose the cell wall, and generate defense signals.^11^ During this process, plants are able to recognize the threat of pathogen infection and initiate a series of defense responses. When plants are damaged, the signal transduction mechanism within the cell is activated, leading to upregulation of PG gene expression and the production of second messenger molecules. These signaling molecules further activate defense-related genes, enhancing the plant’s stress resistance.^12^ Therefore, by regulating the expression level of PG genes and the activity of PG enzymes, plants can quickly respond to external injury stimuli, initiate defense responses, and protect themselves.^13^ Research has shown that after knocking out the *Vmpg7* and *Vmpg8* genes of the apple rot pathogen (*Valsa mali*) and obtaining corresponding mutant strains, these mutant strains exhibit significantly reduced pathogenicity when inoculated into apple branches and leaves.^14^

The factors that interact with *Ca*Las in citrus and their contributions to pathogen virulence are not fully understood, largely because the pathogens are difficult to culture.^15^ As a result, studying resistance genes could be essential for understanding the pathogenesis associated with *Ca*Las.^16^ Additionally, these genes may help develop new control strategies and strengthen the ability of citrus to withstand HLB.^17^

At present, research on the role of PG genes in citrus response to abiotic and biotic stresses is still relatively scarce. On the basis of comparing the results of genomic differential expression analysis,^18^ this experiment screened the *CsPG23* gene that responds to *Ca*Las infection. The expression characteristics of *CsPG23* in citrus were analyzed by qPCR and transient expression techniques. Transient transformation technology and stable genetic transformation technology were used to generate *CsPG23* transgenic hairy roots, and hormone-induced expression analysis was conducted to explore their response patterns to hormones related to biological stress signaling pathways. Yeast two hybrid experiments were used to preliminarily validate the interaction protein of CsPG23, and transgenic hairy roots with the interaction protein were obtained. In summary, this study provided potential candidate genes for molecular breeding of citrus against HLB and theoretical support for disease-resistant breeding practice.

## Materials and methods

### Plant materials, microbial strains, and growth conditions

The experimental materials were taken from the citrus orchard in Wanzhou District, Chongqing. After vacuum infiltration treatment, citrus seedlings were placed in a medium containing vermiculite and grown in a constant temperature incubator at 26 ℃. The light intensity is set to 45 lmol m^−2^s^−1^, and the light duration is 16 h/d. *Nicotiana benthamiana* are cultivated in a light incubator with a temperature maintained between 25 ℃ and 28 ℃. The DH5α strain was grown on Luria Bertani (LB) medium at 37 ℃. Agrobacterium strains EHA105, K599 and GV3101 were cultured on LB medium with 50 µg/mL kanamycin at 28 ℃.

### Cloning and bioinformatics analysis of CsPG23 gene

Total RNA was isolated from the mature leaves of citrus plants, and then it was reverse transcribed into cDNA. For extracting citrus RNA, the procedure adhered to the guidelines specified in the EASYspin Plus kit (Aidlab, Beijing, China). To reverse transcribe RNA into cDNA, 1 µg of total RNA was utilized in a 20 µL reaction with the Prime ScriptTM RT Master Mix (TaKaRa, Ojin, Japan). Primers *CsPG23*-F/R was designed based on the *CsPG23* sequence (Supplementary Table S1), and the *CsPG23* gene was amplified using cDNA as a template. PCR products were inserted into pGEM-Teasy (Promega, WI, USA). The PCR was performed in a volume of 50 µL, including 25 µL Primer STAR Max Premix (TaKaRa, Ojin, Japan), 1.5 µL forward, reverse primer (10 mm·L^−1^), 19 µL ddH_2_O and 3 µL cDNA (0.5 × 10^−5^ ng·L^−2^). PCR amplification conditions were as follows: pre-denaturation at 98°C for 3 min, then conducted 35 amplification cycles (each at 98°C for 30 s, 58°C for 30 s, and 72°C for 30 s), and finally extended at 72°C for 3 min. The sequence of *CsPG23* was determined by Sanger sequencing. The coding sequence of *CsPG23* is 1113bp, encoding 371 amino acid residues. The cis acting elements and open reading frames of *CsPG23* gene, physicochemical properties, hydrophilicity/hydrophobicity, subcellular localization, conserved domains, secondary/tertiary structures, transmembrane domains, and signal peptides were analyzed using an online website.

http://bioinformatics.psb.ugent.be/webtools/plantcare/html.

https://www.expasy.org/resources/protparam.

https://web.expasy.org/protscale/.

http://www.ncbi.nlm.nih.gov/Structure/cdd/wrpsb.cgi.

https://npsa-prabi.ibcp.fr/cgi-bin/npsa_automat.pl?page=/NPSA/npsa_sopma.html.

https://swissmodel.expasy.org/interactive.

https://services.healthtech.dtu.dk/services/SignalP-5.0/.

### *Subcellular localization of CsPG23 in* Nicotiana benthamiana

Primers for subcellular localization were designed based on the *CsPG23* sequence (Suplementary Table S1). PCR product was digested with BamHI/SalI enzymes. The pCAMBIA1300-35S-GFP vector treated with the same endonuclease was inserted to construct the CsPG23: GFP fusion gene, which was transformed into *E. coli* DH5α and screened for positive clones by sequencing. And transformed them into GV3101 agrobacterium tumefaciens.^19^ Agroinfiltration was conducted on the leaves of *N. benthamiana* and three days post-inoculation. To determine the localization of the fusion protein, a FV3000 confocal microscope equipped with a UV light source was used to observe (Olympus, Tokyo, Japan).^20^ The experiment underwent three biological replicates and three technical replicates to ensure the reliability of the results.

### Vector construction and citrus transformation

Using the SGN-VIGS website (https://vigs.solgenomics.net/.) to predict the target region of the *CsPG23* interference fragment,^21^ the 680th to 1079th bp of the *CsPG23* gene were predicted as the interference fragment, and the 700th to 1000th bp of the *CsPG23* gene were selected as the target silencing gene region. TRV2*-CsPG23* and P1300GMN-*CsPG23RNAi* primers were designed (Supplementary Table S1), and PCR amplification was performed using pGEM-Teasy containing *CsPG23* interfering fragments as templates. The *Eco*RI/*Bam*HI enzyme digested product was recovered and inserted into the TRV2 and P1300GMNRNAi vectors treated with the same restriction enzyme to construct the TRV2-CsPG23 and P1300GMN-*CsPG23RNAi* vectors. P1300GMN*-CsPG23* and P1300GMN*-CsAGD8* primers were designed (Supplementary Table S1), and used pGEM-Teasy plasmid containing the CDS sequence of *CsPG23* and *CsAGD8* as a template for PCR amplification. The *Bam*HI/*SaI*I enzyme digested product was recovered and inserted into the P13000GMN vector treated with the same restriction enzyme to construct the P1300GMN-CsPG23 and P1300GMN*-CsAGD8* vectors. The recombinant plasmid was transformed into *E. coli* DH5α and positive clones were screened by sequencing.

Introducing the bacterial solution into the plants through vacuum impregnation, cultivating them in the dark until green fluorescence is emitted, transferring the plants to nutrient soil for further cultivation, and identified the transiently expressed plants after 2 months.^22^

Cutting the citrus hypocotyl diagonally, vacuum infiltration the hypocotyl with *Agrobacterium rhizogenes*, and then inserted the immersed hypocotyl into moist vermiculite for 2 months in constant temperature culture.^23^

### RT-qPCR analysis

qPCR primers for *CsPG23* and *CsAGD8* gene were designed using Primer Blast in NCBI (Supplementary Table S1). 12 µL qPCR reaction system: 6 µL 2 × SYBRPRIME qPCR Kit, 4.4 µL ddH_2_O, 0.3 µL 10 mm·L^−1^ forward and reverse real-time primer, and 1 µL cDNA. The qPCR reactions were as follows: 10 min at 95 ℃, 15 s at 95 ℃, 1 min at 62 ℃, 40 cycles. Using symptomatic leaves, healthy leaves, and sour pomelo as references, the relative expression level of *CsPG23* and *CsAGD8* gene was calculated using the 2^−ΔΔCt^ method.^24^ The experiment underwent three biological replicates and three technical replicates to ensure the reliability of the results.

### Measurement of hormone content

We randomly selected three *CsPG23* positive hairy roots identified as positive, mix well, and weigh about 0.1 g of samples for hormone and reactive oxygen species content detection. The determination methods for salicylic acid (SA), methyl salicylate (MeSA), jasmonic acid (JA), and reactive oxygen species (H_2_O_2_) content using plant enzyme linked immunosorbent assay (ELISA) kits (Jiweibio, Shanghai, China),^25^ with three biological replicates and three parallel replicates for each treatment.

### Yeast two-hybrid

Using PPI (https://cn.string-db.org/cgi/network.) online prediction of potential-interacting proteins of *CsPG23* in citrus, it was found that CsAGD8 protein may interact with it. Using the pGEM-T-CsPG23 plasmid as a template, the *CsPG23* gene was amplified using primer BD-CsPG23-F/R (Supplementary Table S1). *CsPG23* was ligated to pGBKT7 bait vector using homologous recombination method and transformed into *Escherichia coli* DH5α, positive clones were identified through PCR amplification and sequencing, and the pGBKT7-CsPG23 plasmid was constructed. The construction of the pGADT7-CsAGD8 prey vector is the same as above.

The pGADT7-CsAGD8 vector was co-transformed with the pGBKT7-CsPG23 plasmid into Y2Hgold yeast. pGBKT7–53 was co-transformed with pGADT7-T, pGBKT7-Lam and pGADT7-T as positive and negative controls, respectively. Taking Y2HGold competent cells on ice and sequentially add pre-cooled bait plasmids, prey plasmids and PEG/LiAc solution, gently mix by suction and agitation, water bath at 30℃ for 90 min (flip 6–8 times every 10 min to mix well). Shaking and incubating at 30℃ for 90 min. Centrifuging for 5 min and discarding the supernatant. The bacterial solution was then coated on DDO/X (SD/-Leu/-Trp/X-α-gal), TDO (SD/-Leu/-Trp/-His), and QDO (SD/-Leu/-Trp/-His/Ade) media. After 5 days, blue single colonies were taken from DDO/X plates and diluted in 20 µL sterile water, extracted 5 µL of diluted bacterial solution and inoculated it onto the same DDO and QDO media for spot plate testing to observe the growth of yeast.

### Statistical analyses

Statistical analyses of all data were conducted in Excel 365, using the Student’s *t*-test to compare differences between the control and samples at a 5% significance level.

## Results

### Subcellular localization and bioinformatics analysis of CsPG23 gene

The predicted cis acting element of *CsPG23* gene shows that the gene has MYB binding site and methyl jasmonate responsive element (Supplementary Figure S1A). The results of the physicochemical properties of CsPG23 protein showed that a theoretical isoelectric point of 5.11, an acidic protein, a lipid solubility index of 32.91, and an instability index of 37.41, indicating that the protein is a stable protein. The prediction of hydrophilicity/hydrophobicity indicates that the hydrophilic region of the CsPG23 protein polypeptide chain is smaller than the hydrophobic region, indicating that the protein is hydrophobic (Supplementary Figure S1B). As shown in Supplementary Figure S1C, the CsPG23 protein does not possess a conserved domain. The secondary structure, tertiary structure, and signal peptide prediction results of CsPG23 protein are shown in Supplementary Figure S1D-F.

**Figure 1.**
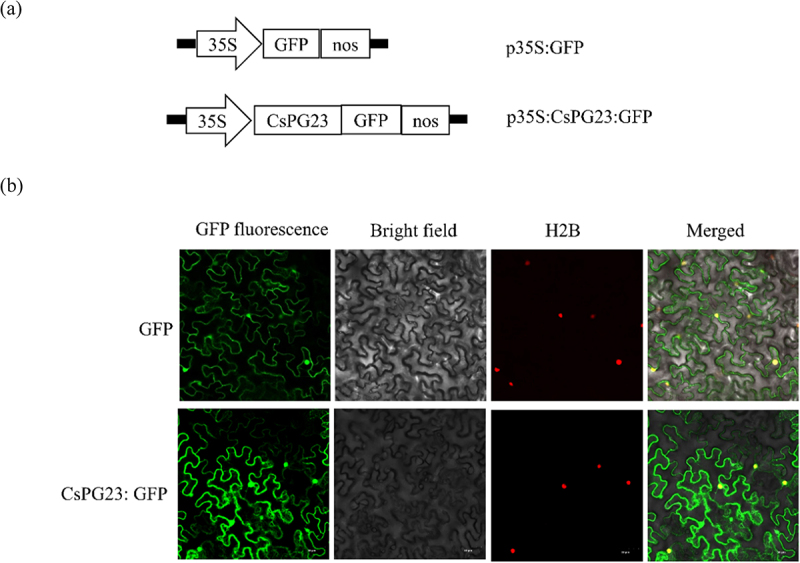
Subcellular location of CsPG23 protein in tobacco epidermis cell. (a) Schematic diagram of the constructs used for agroinfiltration. A 35S, CaMV 35S promoter; NOS, the nopaline synthase terminator. (b) Subcellular localization of CsPG23:GFP fusion protein as observed by confocal microscopy. A histone 2B (H2B) fusion with the red fluorescent protein (RFP) was used as a marker for the nucleus.^20^

CsPG23 coding sequence was fused with the GFP reporter gene (Figure 1(a)). Transient transformation of tobacco was performed using p35S: GFP as a control and H2B: RFP as a nuclear marker gene. Using confocal microscopy, we observed the subcellular localization of CsPG23: GFP fusion protein and found that its distribution within the cell was similar to that of GFP protein alone. Fluorescence signals were detected in the nucleus, cytoplasm, and plasma membrane (Figure 1(b)). The results indicated that

CsPG23 protein is localized in the nucleus, cytoplasm, and cell membrane of tobacco. This experiment underwent three biological replicates and three technical replicates to ensure the reliability of the experimental results.

### Expression of CsPG23 in citrus

**Figure 2.**
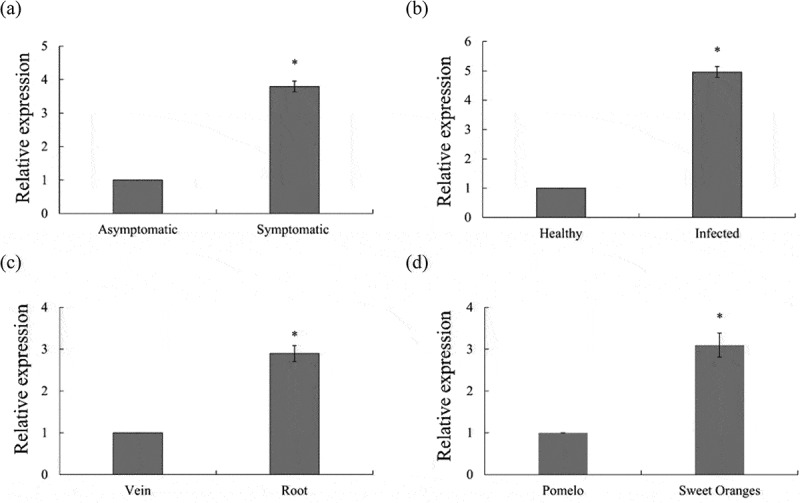
Analysis of the expression characteristics of *CsPG23* gene in citrus. (a) Relative expression of *CsPG23* before and after showing symptoms in sweet oranges. (b) The relative expression level of *CsPG23* in healthy and *Ca*Las-infected sweet oranges. (c) Relative expression levels of *CsPG23* in roots and leaf veins in *Ca*Las-infected sweet oranges. (d) Relative expression of *CsPG23* in *Ca*Las-infected sour pomelo and sweet oranges. Values are expressed as means ± standard deviation of three independent tests. *on top of the bars indicates a significant difference (*p* < 0.05, Student’s *t*-test).

**Figure 3.**
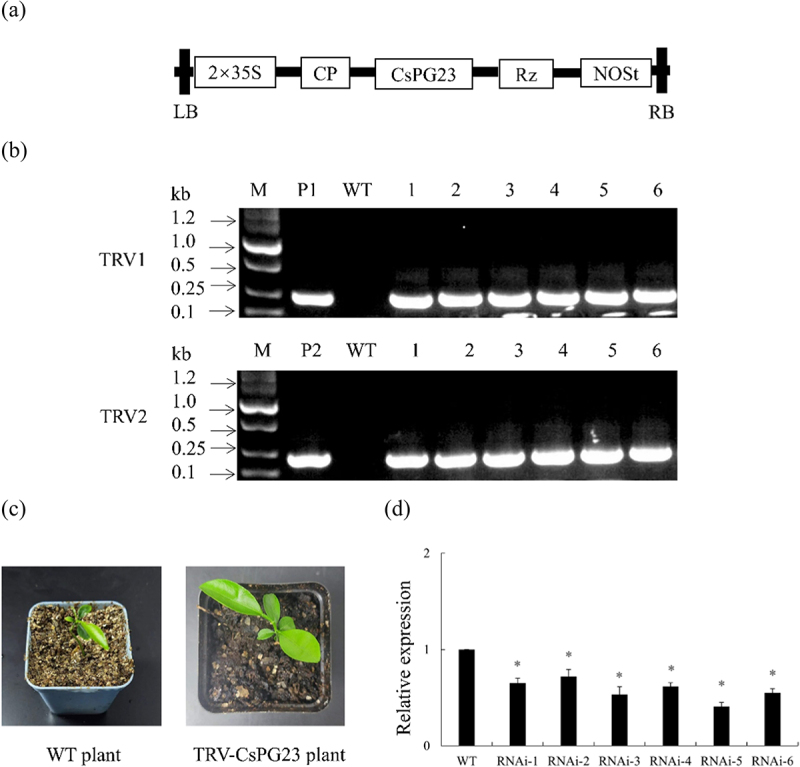
Transgenic plants silencing *CsPG23*. (a) A 35S, 35S promoter; NOSt, the nopaline synthase terminator; LB, left border; RB, right border. (b) Identification of silencing plants by PCR. M, DNA marker; T, TRV plasmid; WT, wild-type control; TRV-#, silencing plants. (c) Phenotypic observation of silencing plants. (d) Relative expression levels of *CsPG23* in silencing plants. Relative expression of *CsPG23* in silencing plants was normalized against its expression in wild-type control using the citrus GAPDH gene^24^ as internal reference. Values are expressed as means ± standard deviation of three independent tests. *on top of the bars indicates significant differences compared to WT control (*p* < 0.05, Student’s *t*-test).

As shown in Figure 3, using the expression level of *CsPG23* in asymptomatic Sweet Oranges leaves as a control, the gene expression level in symptomatic was 3.7 times higher than that in asymptomatic Sweet Oranges leaves (Figure 2(a)). Using the expression level of *CsPG23* in healthy Sweet Oranges leaves as a control, the gene expression level in *Ca*Las-infected Sweet Oranges leaves was upregulated by 4.8 times (Figure 2(b)). Further analysis of the expression characteristics of *CsPG23* in different tissues of Sweet Oranges showed that the expression level of *CsPG23* in roots was 2.8 times higher than that in leaf veins (Figure 2(c)). The differences in metabolites between different citrus varieties may affect the transmission efficiency of psyllids, therefore sour pomelo may have natural resistance potential to citrus HLB.^26^ The expression characteristics of *CsPG23* in different citrus varieties were analyzed using sour pomelo (*Citrus grandis*) with high resistance to HLB and Sweet Oranges with high susceptibility to HLB as experimental materials. The expression level of *CsPG23* in sour pomelo leaves was used as the control, and the gene expression level in Sweet Oranges leaves was upregulated by 2 times (Figure 2(d)). In summary, these results indicated that *Ca*Las infection up-regulated *CsPG23* transcriptional activity. This experiment underwent three biological replicates and three technical replicates to ensure the reliability of the experimental results.

### Generation of TRV-CsPG23 plants

TRV2-*CsPG23* vector was constructed (Figure 3(a)), and positive plants were identified by PCR, resulting in a total of 6 TRV2-*CsPG23* plants (Figure 3(b)). Phenotypic observation revealed that there were no significant differences between TRV2-*CsPG23* plants and WT plants (Figure 3(c)). RNA was extracted from the TRV2-*CsPG23* plants, and the relative expression levels of 6 TRV2-*CsPG23* plants were detected using RT-qPCR. The results showed that the gene expression levels of these plants were significantly lower than those of the WT plants, with RNAi-5 having the lowest expression level, about 0.25 times that of the WT plants (Figure 3(d)).

### Overexpression of CsPG23 in transgenic hairy roots

**Figure 4.**
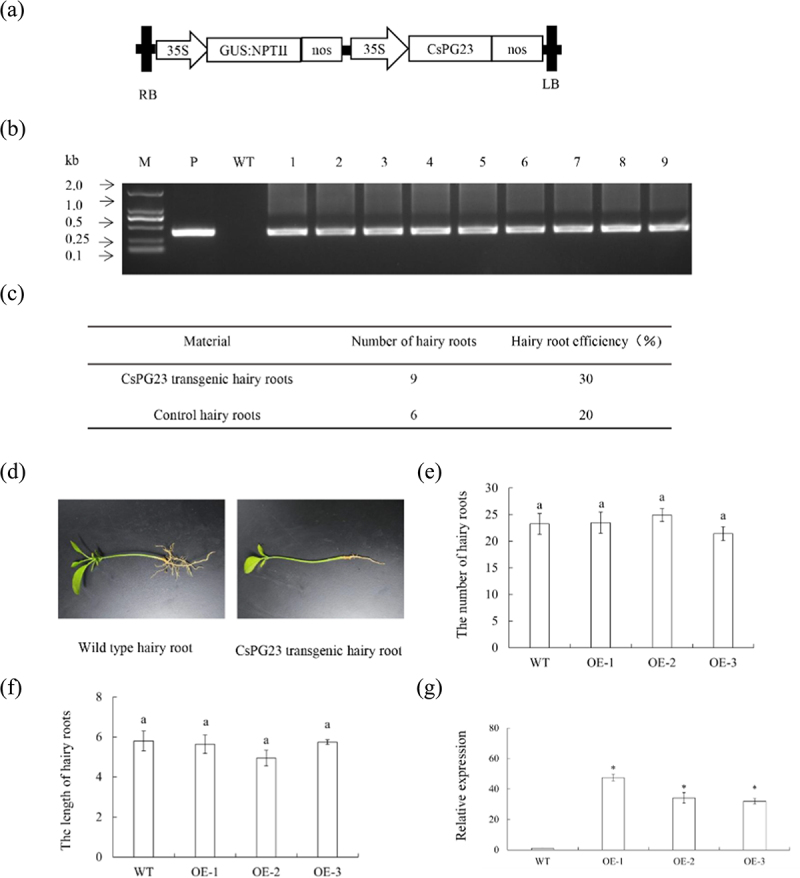
Transgenic hairy roots overexpressing *CsPG23*. (a) A 35S, 35S promoter; NOSt, the nopaline synthase terminator; LB, left border; RB, right border. (b) Identification of transgenic hairy roots by PCR. M, DNA marker; P, p35S: CsPG23 plasmid; WT, wild-type control; OE-#, transgenic hairy roots. (c) Statistics of transgenic hairy roots. (d) Observation on symptoms of transgenic hairy roots. (e) Number statistics of wild-type and transgenic hairy roots. (f) Length statistics of wild-type and transgenic hairy roots. Values are means ± SEMs (*n* = 3). Lowercase letters indicate significant differences among different treatments (*p* < 0.05; Duncan’s multiple comparisons test). (g) Relative expression levels of *CsPG23* in transgenic hairy roots. Relative expression of *CsPG23* in transgenic hairy roots was normalized against its expression in wild-type control using the citrus GAPDH gene^24^ as internal reference. Standard errors were calculated from three hairy roots per line. Values are expressed as means ± standard deviation of three independent tests. *on top of the bars indicates significant differences compared to WT control (*p* < 0.05, Student’s *t*-test).

P1300GMN-*CsPG23* vector was constructed (Figure 4(a)). Nine *CsPG23* transgenic hairy roots were identified by PCR (Figure 4(b)). The rooting rate of transgenic hairy roots was 30% (Figure 4(c)). *CsPG23* transgenic hairy roots are usually white or yellow, with a length of 4 cm to 10 cm (Figure 4(d)). The length and quantity statistics of wild-type and transgenic hairy roots indicate that there is no significant difference in phenotype between *CsPG23* transgenic hairy roots and WT (empty vector) hairy roots (Figure 4(e,f)). Randomly selected three *CsPG23* transgenic hairy roots as one group. Extracting RNA from positive hairy roots and detected the relative expression level of *CsPG23* transgenic hairy roots using RT-qPCR. The results showed that the gene expression level of *CsPG23* transgenic hairy roots was significantly higher than that of the WT group (Figure 4(g)).

### Silencing the expression of CsPG23 in transgenic hairy roots

**Figure 5.**
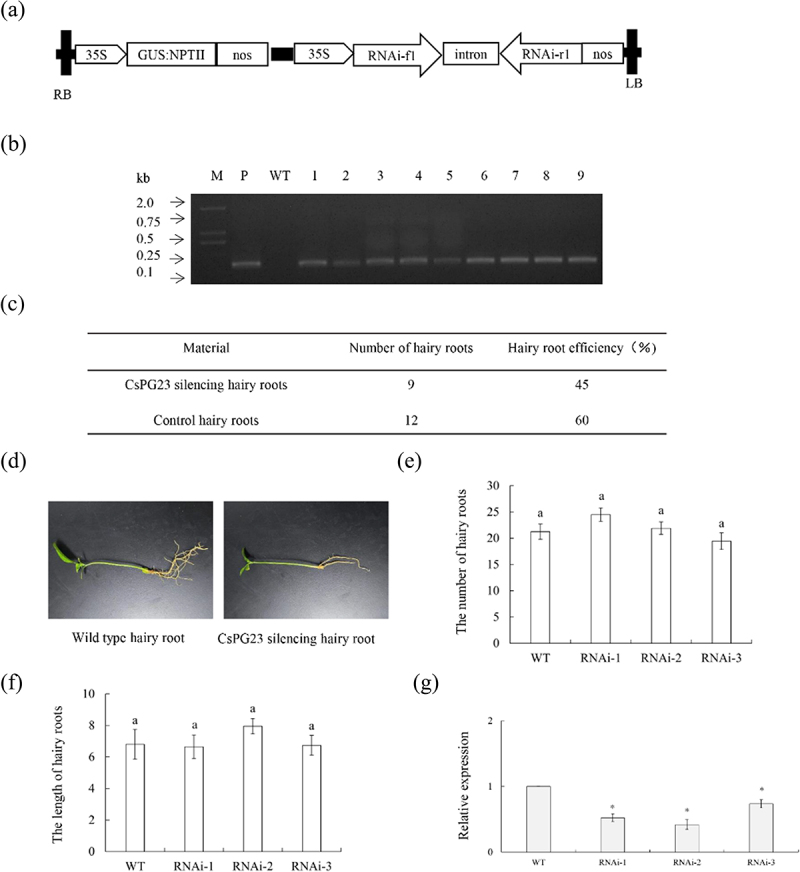
P1300GMN-CsPG23RNAi transgenic hairy roots. (a) A 35S, 35S promoter; NOSt, the nopaline synthase terminator; LB, left border; RB, right border. (b) Identification of transgenic hairy roots by PCR. M, DNA marker; T, P1300GMN-RNAi plasmid; WT, wild-type control; P1300GMN-RNAi #, transgenic hairy roots. (c) Statistics of transgenic hairy roots. (d) Observation on symptoms of transgenic hairy roots. (e) Number statistics of wild-type and transgenic hairy roots. (f) Length statistics of wild-type and transgenic hairy roots. Values are means ± SEMs (*n* = 3). Lowercase letters indicate significant differences among different treatments (*p* < 0.05; Duncan’s multiple comparisons test). (g) Relative expression levels of *CsPg23rnai* in transgenic hairy roots. Relative expression of *CsPg23rnai* in transgenic hairy roots was normalized against its expression in wild-type control using the citrus GAPDH gene^24^ as internal reference. Standard errors were calculated from three hairy roots per line. Values are expressed as means ± standard deviation of three independent tests. *on top of the bars indicates significant differences compared to WT control (*p* < 0.05, student’s *t*-test).

P1300GMN-*CsPG23RNAi* vector was constructed (Figure 5(a)). Nine *CsPG23RNAi* transgenic hairy roots were identified by PCR (Figure 5(b)). The rooting rate of transgenic hairy roots was 45% (Figure 5(c)). Hairy roots are usually white or yellow, with a length of 5 cm to 12 cm (Figure 5(d)). The length and quantity statistics of wild-type and transgenic hairy roots indicate that there is no significant difference in phenotype between *CsPG23RNAi* transgenic hairy roots and WT (empty vector) hairy roots (Figure 5(e,f)). Randomly selected three *CsPG23RNAi* transgenic hairy roots as one group. Extracting RNA from positive hairy roots and detected the relative expression level of *CsPG23RNAi* transgenic hairy roots using RT-qPCR. The results showed that the gene expression level of *CsPG23RNAi* transgenic hairy roots was significantly lower than that of the WT group (Figure 5(g)).

### Characteristics of changes in SA, MeSA, JA and ROS content in CsPG23 transgenic hairy roots

**Figure 6.**
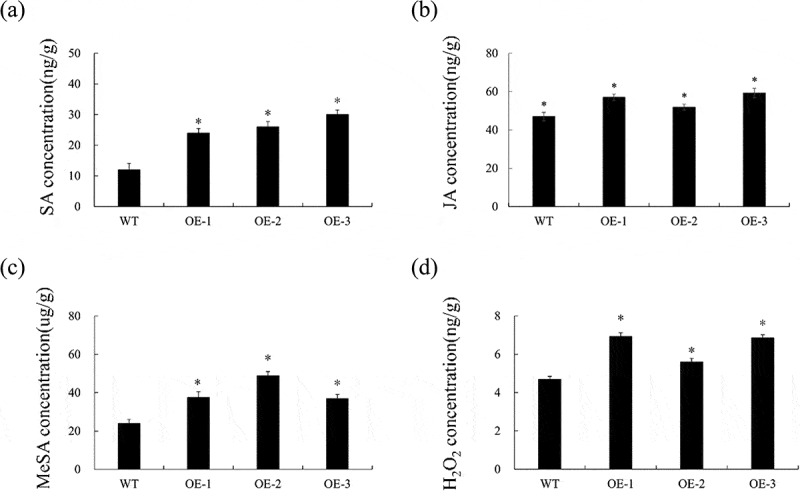
Determination of *CsPG23* transgenic hairy roots hormone content. (a-d) characteristics of SA, JA, MeSA and ROS contents in transgenic hairy roots compared to WT control. Values are expressed as means ± standard deviation of three independent tests. *on top of the bars indicates a significant difference (*p* < 0.05, Student’s *t*-test). WT, wild type; OE-#, transgenic plants.

SA, MeSA, and JA are key signals in plant Systemic Acquired Resistance (SAR) response^25^ and also play an important role in citrus resistance to *Ca*Las infection.^27,28^ To investigate the effect of overexpression of *CsPG23* on SAR response, selected healthy WT, OE-1, OE-2, and OE-3 transgenic hairy roots and measured the changes in SA, MeSA, and JA content. The results showed that compared with the control, the SA, MeSA, and JA levels of *CsPG23* transgenic hairy roots were upregulated (Figure 6(a–c)).

Studies have shown that *Ca*Las infection promoted the accumulation of H_2_O_2_ in hairy roots,^29^ therefore, we detected the changes in H_2_O_2_ content in transgenic hairy roots. In transgenic hairy roots, the H_2_O_2_ content was significantly higher than the control (Figure 6(d)). The results showed that overexpression of *CsPG23* promoted the accumulation of H_2_O_2_. This experiment underwent three biological replicates and three technical replicates to ensure the reliability of the experimental results.

### The expression level of the defense-related genes were remarkably increased in CsPG23 transgenic hairy roots

**Figure 7.**
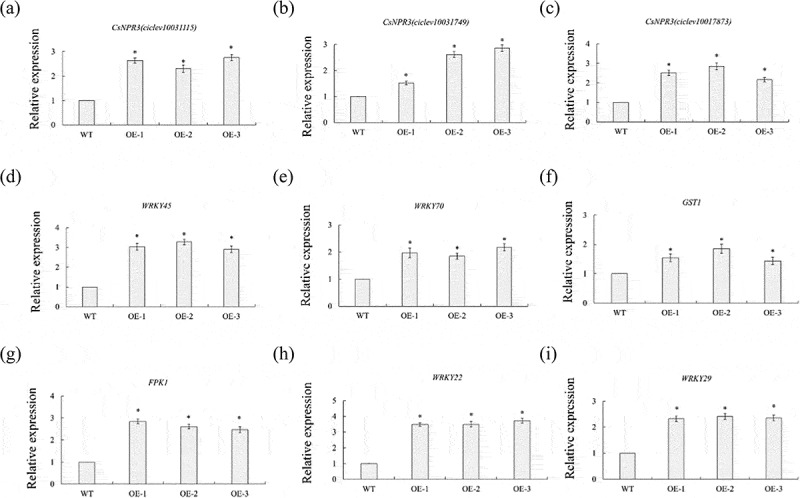
*CsPG23* up-regulated the expression of defense-related genes in transgenic hairy roots. The GAPDH gene was used as an endogenous control. Values are expressed as means ± standard deviation of three independent tests. *on top of the bars indicates significant differences compared to WT control (*p* < 0.05, student’s *t*-test).

The expression of five SAR-associated genes, including three *CsNPR3* genes (Ciclev10017873m, Ciclev10031115m, Ciclev10031749m),^30^ and *CsWRKY45* and *CsWRKY70*^25^ in transgenic hairy roots were investigated by qPCR. The results showed that the expression levels of these genes were significantly increased in *CsPG23* transgenic hairy roots compared with WT hairy roots (Figure 7(a–e)).

The plant immune defense system consists of two mechanisms: Pattern Triggered Immunity (PTI) and effector Triggered Immunity (ETI), which work together to form a dynamic defense network and resist pathogen infection. To further investigate how *CsPG23* involves with plant defense responses, the expression of 4 PTI, ETI- (*CsFRK1*, *CsGST1*, *CsWRKY22* and *CsWRKY29*)^31^ related marker genes were analyzed. The results showed that these genes were significantly increased in *CsNPR3* transgenic hairy roots compared with WT hairy roots (Figure 7(f–i)).

### The identification of proteins interaction

**Figure 8.**
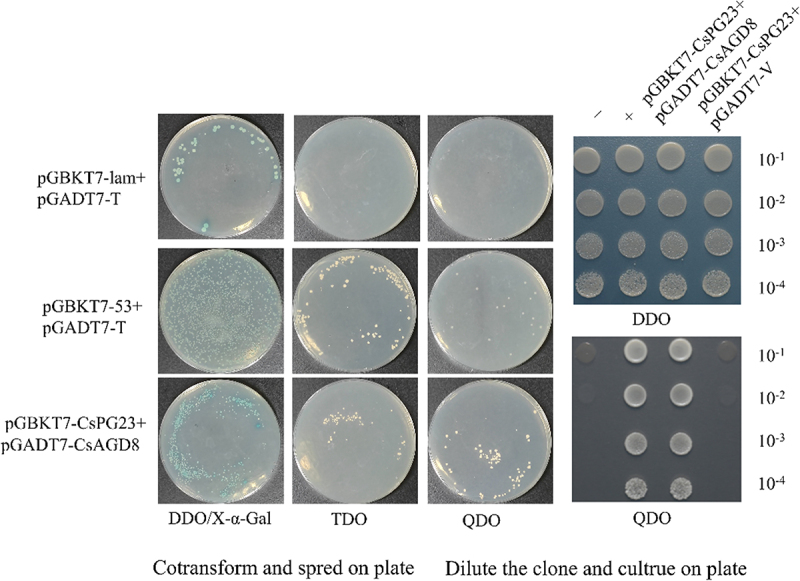
CsPG23 interacts with CsAGD8. Y2H assays show that CsPG23 interacts with AGD8. BD indicates pGBKT7 vector. AD indicates pGADT7 vector. BD-53 + AD-T is a positive control, BD-Lam + AD-T is a negative control.

Performing yeast point-to-point hybridization validation on predicted proteins. Figure 8 shows that the pGADT7-CsAGD8 fusion expression vector plasmid and pGBKT7-CsPG23 plasmid co transformed into Y2Hgold and grew normally on DDO and QDO screening media, indicated a possible interaction between the CsAGD8 protein and CsPG23 protein.

### Generation of transgenic plants overexpressing CsAGD8

**Figure 9.**
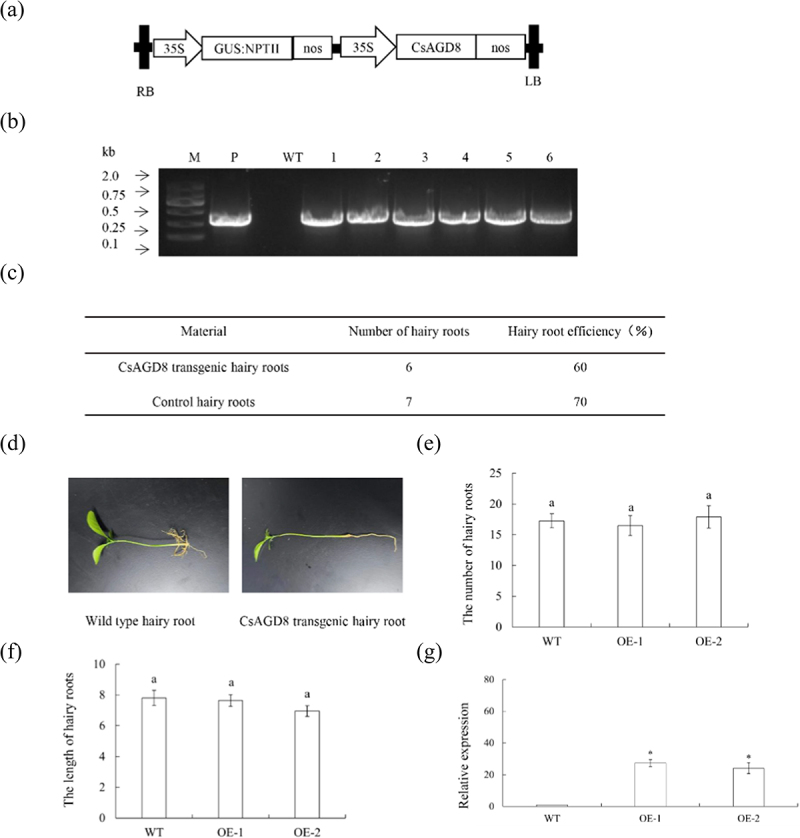
Transgenic plants overexpressing *CsAGD8*. (a) T-DNA structure of plant expression vector for the genetic transformation of citrus. (b) Identification of transgenic plants by PCR. M, DNA marker, P, p35S: CsAGD8 plasmid; WT, wildtype control; OE-#, transgenic plants. (c) Statistics of transgenic hairy roots. (d) Observation on symptoms of transgenic hairy roots. (e) Number statistics of wild-type and transgenic hairy roots. (f) Length statistics of wild-type and transgenic hairy roots. Values are means ± SEMs (*n* = 3). Lowercase letters indicate significant differences among different treatments (*p* < 0.05; Duncan’s multiple comparisons test). (g) Relative expression levels of *CsAGD8* in transgenic plants. Relative expression of *CsAGD8* in transgenic plants was normalized against its expression in wild-type control using the citrus GAPDH gene^24^ as internal reference. Values are expressed as means ± standard deviation of three independent tests. *on top of the bars indicates significant differences compared to WT control (*p* < 0.05, student’s *t*-test).

We further investigated the biological function of CsAGD8 protein. A plant overexpression vector P1300GMN-*CsAGD8* vector was constructed (Figure 9(a)). Identification of plants by PCR resulted in a total of 6 *CsAGD8* transgenic hairy roots (Figure 9(b)). The rooting rate of transgenic hairy roots was 60% (Figure 9(c)). Hairy roots are usually white or yellow, with a length of 8 cm to 11 cm (Figure 9(d)). The length and quantity statistics of wild-type and transgenic hairy roots indicate that there is no significant difference in phenotype between *CsAGD8* transgenic hairy roots and WT (empty vector) plants (Figure 9(e,f)). Randomly selected three *CsAGD8* transgenic hairy roots as one group. RNA was extracted from the positive hairy roots, and the relative expression levels of *CsAGD8* transgenic plants were detected using RT-qPCR. The results showed that the gene expression levels of *CsAGD8* transgenic plants were significantly higher than those of the WT (Figure 9(g)).

### Generation of transgenic hairy roots silencing CsAGD8

**Figure 10.**
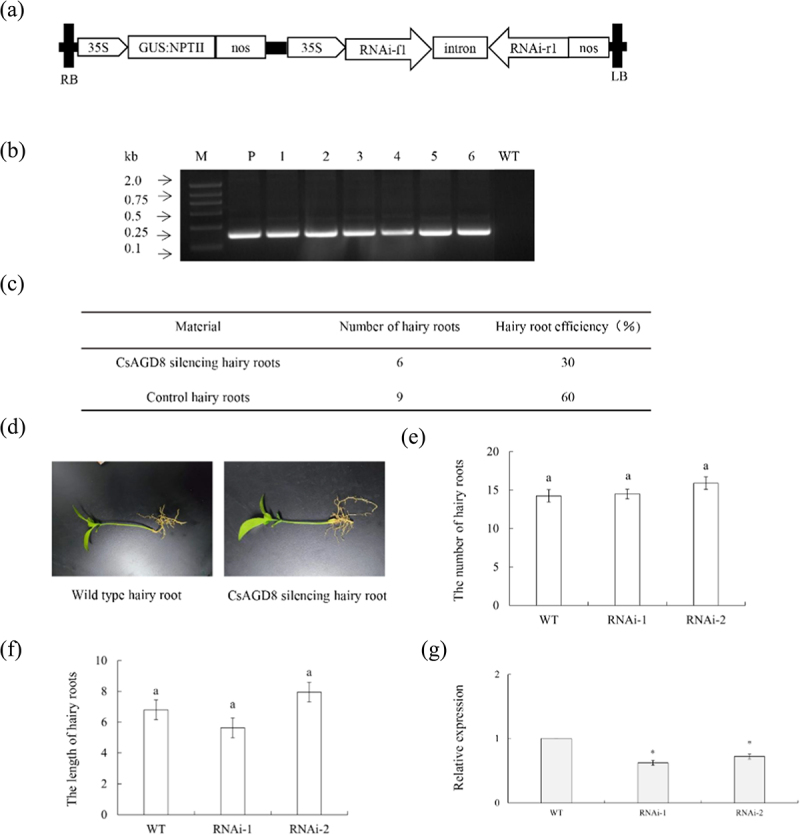
P1300GMN-CsAGD8RNAi transgenic hairy roots. (a) A 35S, 35S promoter; NOSt, the nopaline synthase terminator; LB, left border; RB, right border. (b) Identification of transgenic hairy roots by PCR. M, DNA marker; T, P1300GMN-RNAi plasmid; WT, wild-type control; P1300GMN-RNAi #, transgenic hairy roots. (c) Statistics of transgenic hairy roots. (D) Observation on symptoms of transgenic hairy roots. (e) Number statistics of wild-type and transgenic hairy roots. (f) Length statistics of wild-type and transgenic hairy roots. Values are means ± SEMs (*n* = 3). Lowercase letters indicate significant differences among different treatments (*p* < 0.05; Duncan’s multiple comparisons test). (g) Relative expression levels of *CsAgd8rnai* in transgenic hairy roots. Relative expression of *CsAgd8rnai* in transgenic hairy roots was normalized against its expression in wild-type control using the citrus GAPDH gene^24^ as internal reference. Standard errors were calculated from three hairy roots per line. Values are expressed as means ± standard deviation of three independent tests. *on top of the bars indicates significant differences compared to WT control (*p* < 0.05, student’s *t*-test).

P1300GMN-Cs*AGD8RNAi* vector was constructed (Figure 10(a)). Six *CsAGD8RNAi* transgenic hairy roots were identified by PCR (Figure 10(b)). The rooting rate of transgenic hairy roots was 30% (Figure 10(c)). Hairy roots are usually white or yellow, with a length of 6 cm to 12 cm (Figure 10(d)). The length and quantity statistics of wild-type and transgenic hairy roots indicate that there is no significant difference in phenotype between *CsAGD8RNAi* transgenic hairy roots and WT (empty vector) plants (Figure 10(e,f)). Randomly selected three *CsAGD8RNAi* transgenic hairy roots as one group. Extracting RNA from positive hairy roots and detected the relative expression level of *CsAGD8RNAi* transgenic hairy roots using qPCR. The results showed that the gene expression level of *CsAGD8RNAi* transgenic hairy roots was significantly lower than that of the WT group (Figure 10(g)).

## Discussion

In-depth research on the pathogenic process of pathogens on host plants can help understand the mechanism of disease occurrence, among which the smooth invasion of pathogens into plants and their ability to colonize and grow in plants are the primary prerequisites for pathogens to further exert their effects.^32^ During the process of pathogen infection in plants, PG can effectively destroy the structure of the cell wall by degrading the galacturonic acid in the intermediate layer of the primary wall and the intercellular layer, weakening the strength and integrity of the cell wall.^33^ Pathogens can easily penetrate the plant cell wall and quickly invade the interior of the cell, leading to the occurrence of diseases.^34^

PG is involved in multiple physiological processes such as organ shedding, anther dehiscence, and pollen maturation.^35^ In-depth research has been conducted on PG in model plants such as Arabidopsis, tomato, and rice, providing important theoretical support for our understanding of the role of PG in plant disease resistance response.^36^ However, the specific function of PG in citrus disease resistance is still unclear and requires further exploration. Therefore, in-depth study of the function of PG in citrus can not only reveal its mechanism of action in plant disease resistance response but also provide a solid theoretical basis for developing new disease-resistant varieties and formulating effective disease prevention and control strategies.^37^

This study screened and cloned the citrus PG gene *CsPG23* through transcriptome data. CsPG23 contains 371 amino acid residues, and the sequence within its domain is highly conserved. Subcellular localization analysis suggests that CsPG23 protein may regulate the transcriptional activity of genes related to the cell wall in the nucleus, thereby regulating cell wall metabolism.

In the process of interaction between pathogens and plants, transcriptional expression analysis of endogenous genes helps to analyze the defense response during pathogen infection.^38^ The differential expression of endogenous genes in citrus fruits at different times and spaces may be key to pathogen invasion and colonization, as well as host tolerance and survival.^39^ In this study, we found that the expression level of *CsPG23* was significantly elevated in symptomatic leaves. This result suggested that *CsPG23* may be closely related to the plant’s response to HLB. Meanwhile, compared with healthy leaves, the expression level of *CsPG23* in *Ca*Las-infected leaves was significantly higher, further supporting this viewpoint. Further analysis was conducted on the expression levels of *CsPG23* in the leaves of sour pomelo and Sweet Oranges, and it was found that the expression level of *CsPG23* was significantly upregulated in the leaves of Sweet Oranges. The expression level of *CsPG23* gene in the roots of Sweet Oranges is significantly higher than that in the leaf veins, suggesting that *CsPG23* may play an important role in the colonization of *Ca*Las in the roots. In summary, the results indicated that *CsPG23* expression was associated with variety tolerance, tissue location, and symptom development.

The process of genetic transformation in citrus, facilitated by Agrobacterium tumefaciens, faces several challenges, including lengthy cycles, high difficulty levels, occurrences of false positives, and susceptibility to contamination, which significantly obstruct the progress of citrus genetic breeding research.^40^ Moreover, the lengthy onset period of HLB disease, coupled with the complexities in assessing resistance, considerably impedes the advancement of studies concerning citrus HLB.^41^ Irigoyen et al.^42^ demonstrated that hairy roots supported the accumulation of *Ca*Las. They used hairy roots for antibacterial screening and discovered multiple genetic and chemical methods that effectively inhibited *Ca*Las in plant tissues.^42^ In this study, citrus hypocotyls were used as materials to obtain overexpressing *CsPG23* transgenic hairy roots after vacuum infiltration with Agrobacterium rhizogenes. Hairy roots are an important tool for studying plant root biology and secondary metabolites and may have good effects on both woody and medicinal plants.^43^ In this study, the method of using Agrobacterium rhizogenes mediated citrus hypocotyls and produced hairy roots was used to avoid bacterial contamination, efficiently produced transgenic hairy roots, and quickly performed preliminary functional identification and analysis of genes, significantly shortened the cycle of citrus genetic transformation and resistance evaluation, provided a good foundation for citrus genetic breeding.^44^ VIGS has shown potential application prospects in the study of plant gene function. VIGS is a powerful tool for inhibiting endogenous gene expression, and its silencing mechanism is similar to RNAi.^45^ Because VIGS has the advantages of fast, efficient, high-throughput, convenience, and affordability, it has been used for gene function research in various plants such as potatoes, native tobacco,^46^ Arabidopsis,^47^ tomatoes.^48^ This study constructed a citrus TRV-mediated VIGS gene silencing system and a hairy root system mediated by Agrobacterium rhizogenes, which can be widely used for rapid gene function research in most herbaceous and woody plants.

When plants are invaded by pathogens, they can quickly activate a series of complex disease resistance mechanisms.^49^ The basis of these mechanisms is the disease resistance signaling pathway, in which SA, JA, and ROS play crucial roles as key molecules.^50^ When plants detect the invasion of pathogens, SA and JA are rapidly synthesized and transmit signals, inducing a series of corresponding immune responses in plant cells. Meanwhile, ROS also serve as signaling molecules and play an important role in plant immune processes.^51^ In this signaling network, PG family genes play a crucial role. Research has shown that these genes are involved in plant immune defense mediated by SA, JA, and ROS signaling, helping plants establish strong disease resistance.^52^ In addition, research on the tolerance of citrus to HLB has found a significant correlation between high levels of SA and JA and the disease resistance of citrus trees. The high levels of SA and JA are not only important indicators of plant defense, but also key factors in enhancing citrus tolerance to HLB.^53^ In this study, the results showed that hormone levels such as SA, JA, and ROS were significantly upregulated and overexpression of *CsPG23* significantly affected the expression of related genes in the PTI and ETI pathways in transgenic hairy roots. Based on this, it is indicated that *CsPG23* may be related to HLB tolerance and may be involved in the process of citrus resistance to *Ca*Las infection.

PG enzymes exhibit a dual role in the interaction between plants and pathogens: on the one hand, they promote pathogen infection by degrading cell wall pectin, and on the other hand, they activate plant immunity by releasing damage associated molecular patterns (DAMPs) such as oligogalacturonic acid.^54^ The double-edged sword characteristic of PG enzyme reflects the complexity of plant-pathogen interaction: pathogens invade through enzymatic hydrolysis, while plants activate defense by hijacking their products (such as DAMP).^55^ In the future, it is necessary to combine structural biology (such as PGIP-PG complex analysis) and gene editing technology to accurately regulate signal balance and provide new ideas for disease-resistant breeding.^56^

Investigating the relationships among proteins holds considerable importance for understanding disease mechanisms.^57^ In this research, a target protein that interacts with CsPG23 was identified through predictive, and the interaction between CsPG23 and CsAGD8 was subsequently validated using Y2Hgold. *AGD8* is involved in the recruitment of Arf1 GDP to the Golgi apparatus in *Arabidopsis thaliana*.^58^ RNAi interference with low-level expression of *AGD8* in plants exhibits significant physiological and cytological abnormalities. The Golgi apparatus of these plants exhibits abnormal morphology, revealing structural disorder.^59^ In addition, the efficiency of protein transport is inhibited, leading to significant growth and development arrest in the plants. Relatively speaking, high-level expression of *AGD8* exhibits a promoting effect, especially in the Golgi apparatus region, which facilitates the accumulation of Arf1. This accumulation effectively suppressed the Golgi apparatus damage and inhibition of vacuolar transport caused by overexpression of *AGD7*, further supporting the important role of *AGD8* in intracellular transport mechanisms.^60^ In plants, the physiological role of AGD8 protein has been studied through mutation and overexpression methods, and the physiological functions of some *AGD* isoforms have been confirmed through their mutant phenotypes: defects in vascular formation, flower organ shedding, and root hair and pollen tube growth.^61^ The AGD8 gene belongs to the ADP ribosylation factor GTPase activating protein (ArfGAP) family and may be involved in regulating membrane remodeling or cargo sorting in Golgi vesicle transport. *PG23* may be related to the synthesis or modification of cell wall polysaccharides. If its products are transported through the Golgi apparatus, *AGD8* may indirectly affect the function of *PG23* by regulating vesicle formation. *SEC14L2* vesicles derived from Golgi apparatus regulate endocytic division through phosphatidylinositol (PtdIns) metabolism (PtdIns4P → PtdIns3P transition).^62^ Similar mechanisms may extend to other membrane systems, such as cell wall precursor transport. If *AGD8* is involved in this process, there may be signal cross-over with *PG23* mediated cell wall synthesis.^62^ It is indicated that *CsAGD8* may be involved in physiological processes such as vesicular and protein transport in citrus.

## Conclusions

This experiment analyzed the expression characteristics of *CsPG23* in citrus and conducted preliminary interaction verification on the predicted protein CsAGD8. Using Agrobacterium rhizogenes-mediated genetic transformation technology, *CsPG23* and *CsAGD8* transgenic hairy roots were obtained. Hormone content detection and immune defense-related gene analysis were performed on the transgenic hairy roots to further explore the roles of *CsPG23* and *CsAGD8* in the infection process of citrus HLB.

## Supplementary Material

Supplementary Table 1.xlsx

Supplementary figures.docx

## Data Availability

The datasets generated during and/or analyzed during the current study are available from the corresponding author on reasonable request.
