# Supplementary material for: Expression characteristics of CsPG23 in citrus and analysis of its interacting protein
Source: Plant Signal Behav. 2025 May 22;20(1):2508418. doi: 10.1080/15592324.2025.2508418 (PMC12101599; doi:10.1080/15592324.2025.2508418)
Supplement: Supplementary figures.docx [file KPSB_A_2508418_SM4834.docx]

**Expression characteristics of *CsPG23* in citrus and analysis of its interacting protein**

Qing He^a^, Xiao He^a,^*

^a^ Chongqing Three Gorges Medical College, Chongqing, People’s Republic of China

* Corresponding authors. E-mail addresses: 2789874475@qq.com

**
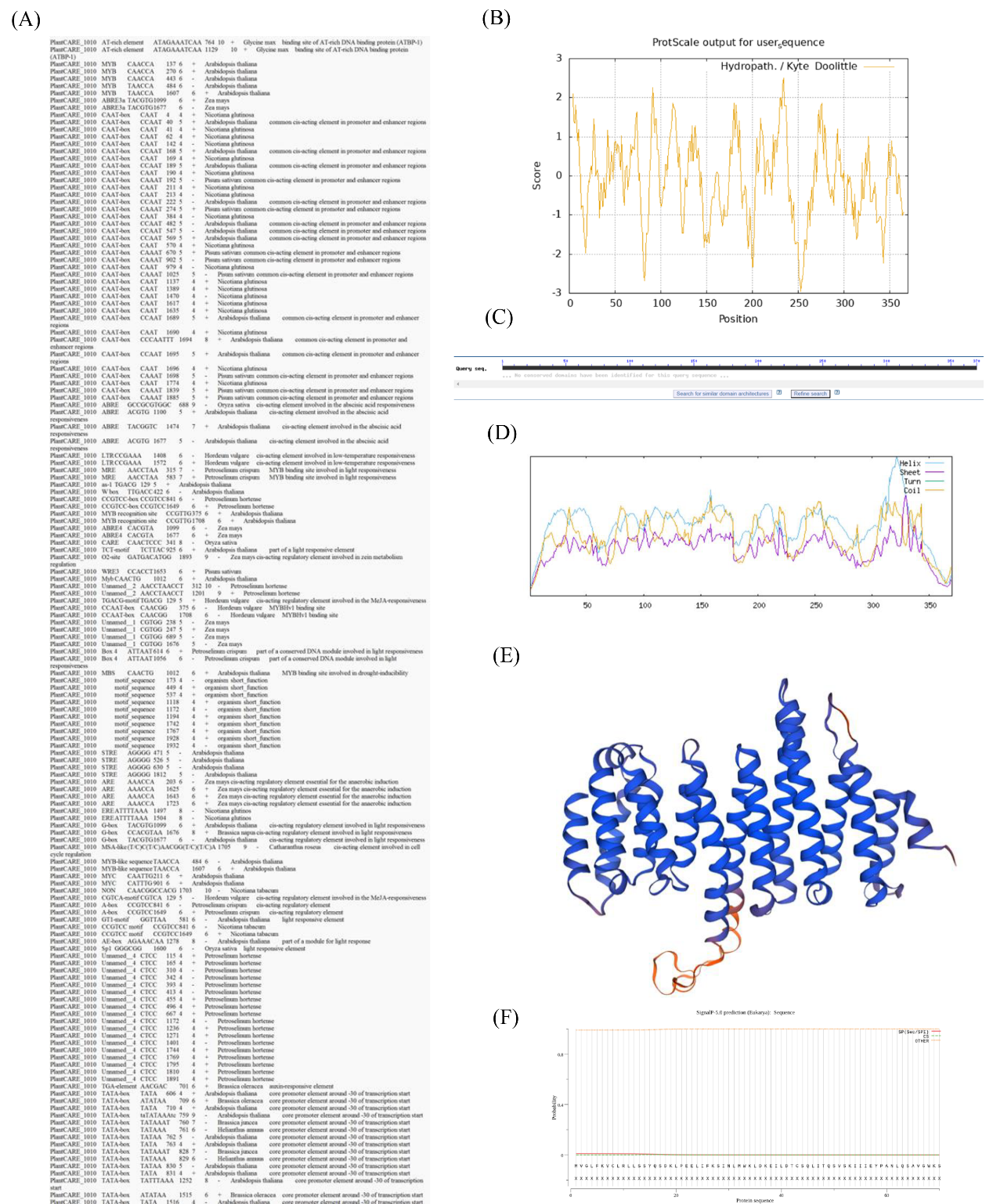
**

**Supplementary Figure 1.** **Bioinformatics analysis of *CsPG23* gene. (A)** Relevant information of the core elements in *CsPG23* promoter sequence. **(B)** Hydrophilicity/hydrophobicity analysis of CsPG23 amino acid sequence. **(C)** Conserved domains of CsPG23 protein. **(D)** The secondary structure of CsPG23 protein. **(E)** The tertiary structure of CsPG23 protein. **(F)** Prediction of signal peptides for CsPG23 protein.
